# Retraction Note to: Synthesis, characterization and in vitro studies of doxorubicin-loaded magnetic nanoparticles grafted to smart copolymers on A549 lung cancer cell line

**DOI:** 10.1186/s12951-018-0434-2

**Published:** 2019-01-19

**Authors:** Abolfazl Akbarzadeh, Mohammad Samiei, Sang Woo Joo, Maryam Anzaby, Younes Hanifehpour, Hamid Tayefi Nasrabadi, Soodabeh Davaran

**Affiliations:** 1 Department of Medical Nanotechnology, Faculty of Advanced Medical Science, Tabriz University of Medical Sciences, Tabriz, Iran; 2 Department of Endodontics, Dental School, Tabriz University of Medical Sciences, Tabriz, Iran; 3 School of Mechanical Engineering, WCU Nanoresearch Center, Yeungnam University, Gyeongsan, 712-749 South Korea

## Retraction Note to: J Nanobiotechnol (2012) 10: 46 10.1186/1477-3155-10-46

The Editors have retracted this article [1] because Fig. 6a and c have been used in three other publications to represent scanning electron micrographs of different nanoparticles [2–4]. The data reported in this article are therefore unreliable. In addition, Fig. 3 was reproduced from [5] with retrospective permission and the credit line should read as follows: “Reprinted from Acta Biomaterialia, Volume 3, Zhang, J. and Misra, R.D.K., Magnetic drug-targeting carrier encapsulated with thermosensitive smart polymer: core–shell nanoparticle carrier and drug release response, pp. 838–850, copyright (2007) with permission from Acta Materialia Inc. Authors Abolfazl Akbarzadeh, Maryam Anzaby, Soodabeh Davaran, Sang Woo Joo and Mohammad Samiei agree to this retraction. Authors Younes Hanifehpour and Hamid Tayefi Nasrabadi have not responded to any correspondence about this retraction.
